# Reply to Larsen and Riisgård: Fluid dynamics of choanocyte chambers

**DOI:** 10.1073/pnas.2507566122

**Published:** 2025-05-30

**Authors:** Takumi Ogawa, Shuji Koyama, Toshihiro Omori, Kenji Kikuchi, Hélène de Maleprade, Raymond E. Goldstein, Takuji Ishikawa

**Affiliations:** ^a^Department of Finemechanics, Tohoku University, Sendai 980-8579, Japan; ^b^Sorbonne Université, CNRS Institut Jean Le Rond d’Alembert, Paris F-75005, France; ^c^Department of Applied Mathematics and Theoretical Physics, University of Cambridge, Cambridge CB3 0WA, United Kingdom; ^d^Department of Biomedical Engineering, Tohoku University, Sendai 980-8579, Japan

In their comment (1), Larsen and Riisgård (LR) question the modeling assumptions made in our paper (2), the first computational study of interacting flagella in the spherical geometry of choanocyte. LR focus on the interaction of flagellar vanes with the collar in establishing the pressure drop across each choanocyte. We acknowledged such structures in some sponges and their possible role in the fluid mechanics but noted that in our studies of *Ephydatia muelleri*, we did not see them and chose to focus on the simplest setting to explore the role of chamber geometry.

In early work (3), LR investigated the pressure drop Δ*p* that exists between the pores on surface of sponges and the central channel out of which flows fluid from the sponge interior and found Δ*p* ∼ 2.7 mm H_2_O. From morphological evidence, they concluded that choanocyte chambers are in parallel and assigned Δ*p* to a single chamber. Computational studies (4) of a choanocyte with prescribed flagellar motion and a vane that fits rather tightly in the collar led to the hypothesis that Δ*p* is associated with a single choanocyte, which functions as a pump with leakage through the meshlike structure around the basal part of the flagellum. In units appropriate to the picoNewton (pN) forces *F* of flagella and the *μ*m size of choanocytes, using *F* = Δ *pA* where *A* is the cross-sectional area of the basal structure, 2.7 mm H_2_O in their computational cross-sectional area of 9.6 *μ*m^2^ corresponds to *F* = 254pN. Since *F* is hypothesized to arise from the fraction of the flagellum within the collar, ranging from ≲1/3 to 2/3, this implies flagellum exerts a force of ∼400 to 750 pN. We are not aware of any direct measurements of force generation by choanocyte flagella, but studies of the swimming of choanoflagellates (5) found *F* ∼ 10pN. Since flagella may respond to higher loads with higher forces, this discrepancy by a factor of 40 to 75 does not immediately rule out the hypothesis that a single flagellum generates the observed Δ*p*, but it suggests that the matter of how the many choanocytes within a chamber contribute to Δ*p* is not yet settled. A recent study (6) finds the vane width is at most half of the collar diameter, suggesting the gasket effect would be significantly reduced.

From a fluid dynamical point of view, the leaky-collar model corresponds to a fluid acted on by a distributed set of Stokeslets along the length of each flagellum. This is a variant of the problem that we have studied; an obvious next step would be to merge these two approaches to understand the full dynamics of interacting choanocytes in the spherical geometry of sponge choanocyte chambers. At the same time, this discussion highlights the need to develop further the experimental approaches to quantifying the forces involved in choanocyte fluid mechanics.
